# Supplementary figures and images for: A homogeneous time-resolved fluorescence screen to identify SIRT2 deacetylase and defatty-acylase inhibitors
Source: PLoS One. 2024 Jun 24;19(6):e0305000. doi: 10.1371/journal.pone.0305000 (PMC11195995; doi:10.1371/journal.pone.0305000)

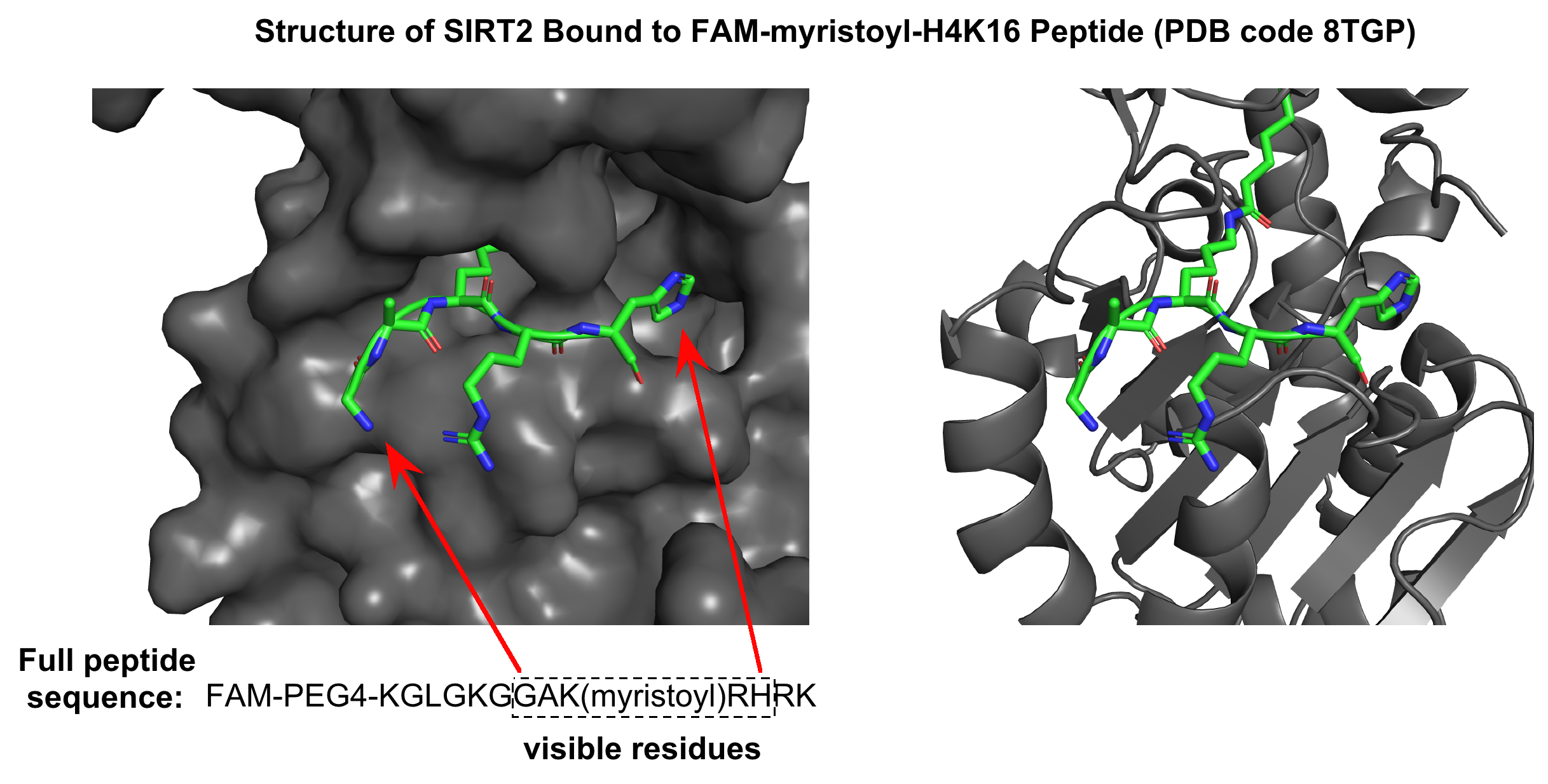

Supplement: S1 Fig — The panels show surface or cartoon representations of SIRT2 colored dark gray from the same view, and the bound peptide was shown in sticks and colored by atom (carbon, green; nitrogen, blue; oxygen, red). Only five amino acid residues from the myristoylated peptide were visible in the crystal structure, as indicated. (TIF) [file pone.0305000.s001.tif]

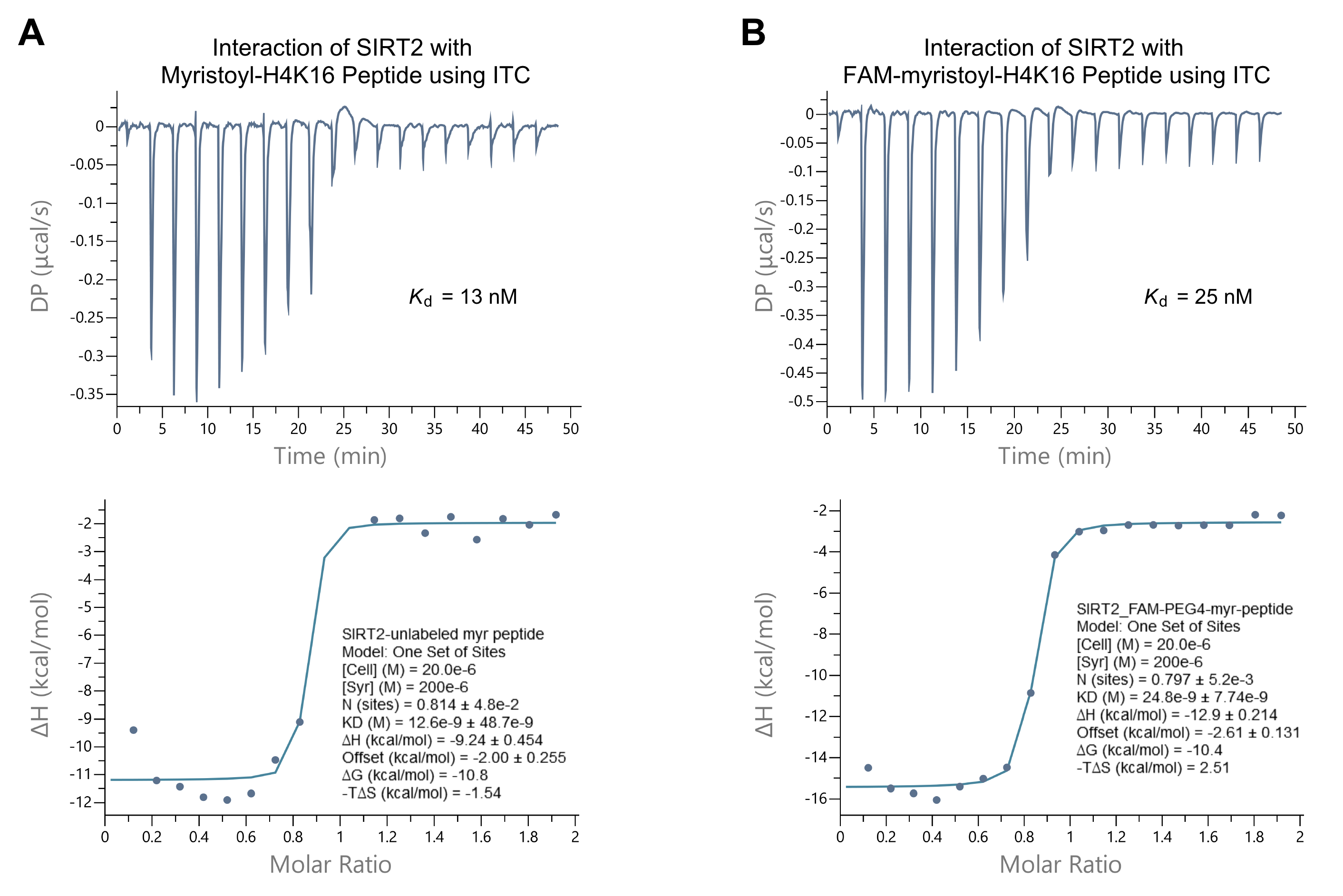

Supplement: S2 Fig — (A) Binding isotherm and fit curve determining a Kd of 13 nM for SIRT2’s interaction with an unlabeled myristoyl-H4K16 peptide. (B) Binding isotherm and fit curve determining a Kd of 25 nM for SIRT2’s interaction with FAM-myristoyl-H4K16 peptide. ITC was performed in PBS using a Malvern Panalytical MicroCal PEAQ-ITC. The sample cell and syringe were set to 25°C. 20 μM SIRT2 was placed in the sample cell, and 200 μM peptide was placed in the ITC syringe. 19 injections of peptide were automatically applied to the sample cell in a 2 μl volume, except for the first injection (0.4 μl). A one site binding model was fit to the data by the software to calculate binding parameters. (TIF) [file pone.0305000.s002.tif]

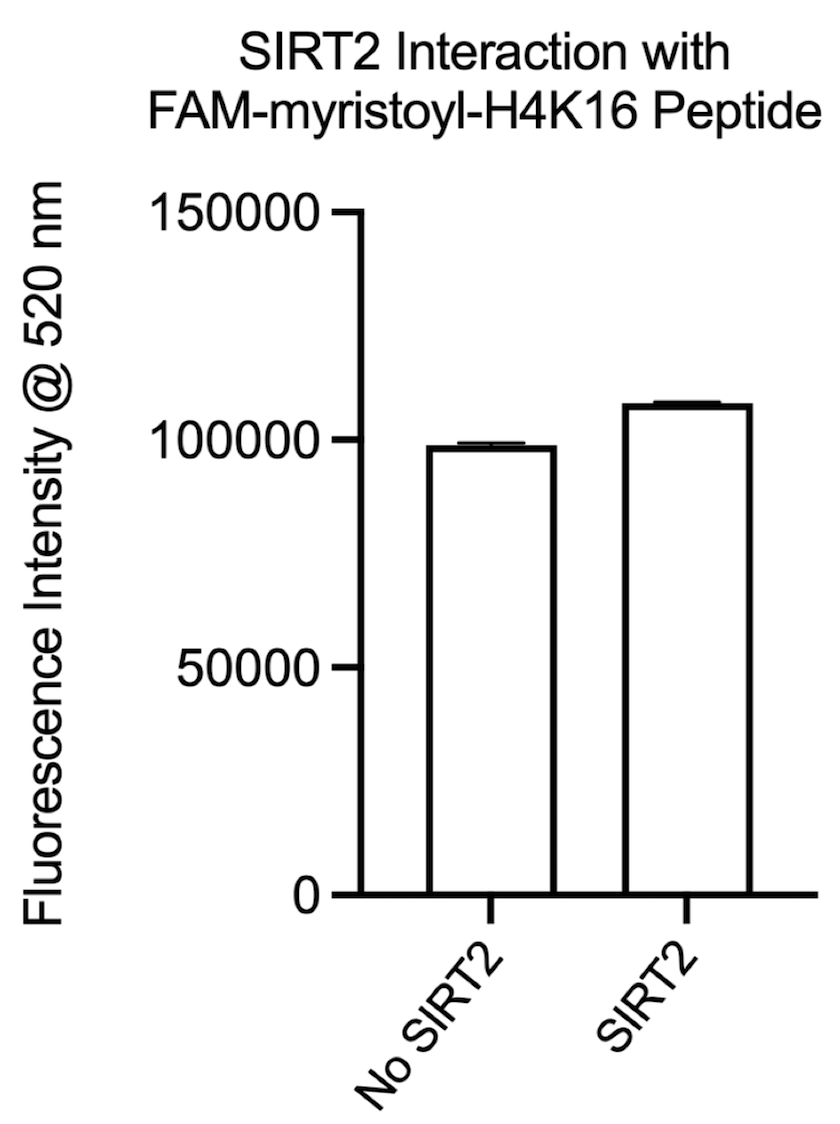

Supplement: S3 Fig — In this experiment, the fluorescence of 50 nM FAM-myristoyl-H4K16 peptide was measured in the presence or absence of 1 μM SIRT2 (excitation/emission wavelengths were 340 nm/520 nm, similar to HTRF assays). The fluorescence intensity differed by only 9.5% between the two conditions. (TIFF) [file pone.0305000.s003.tiff]

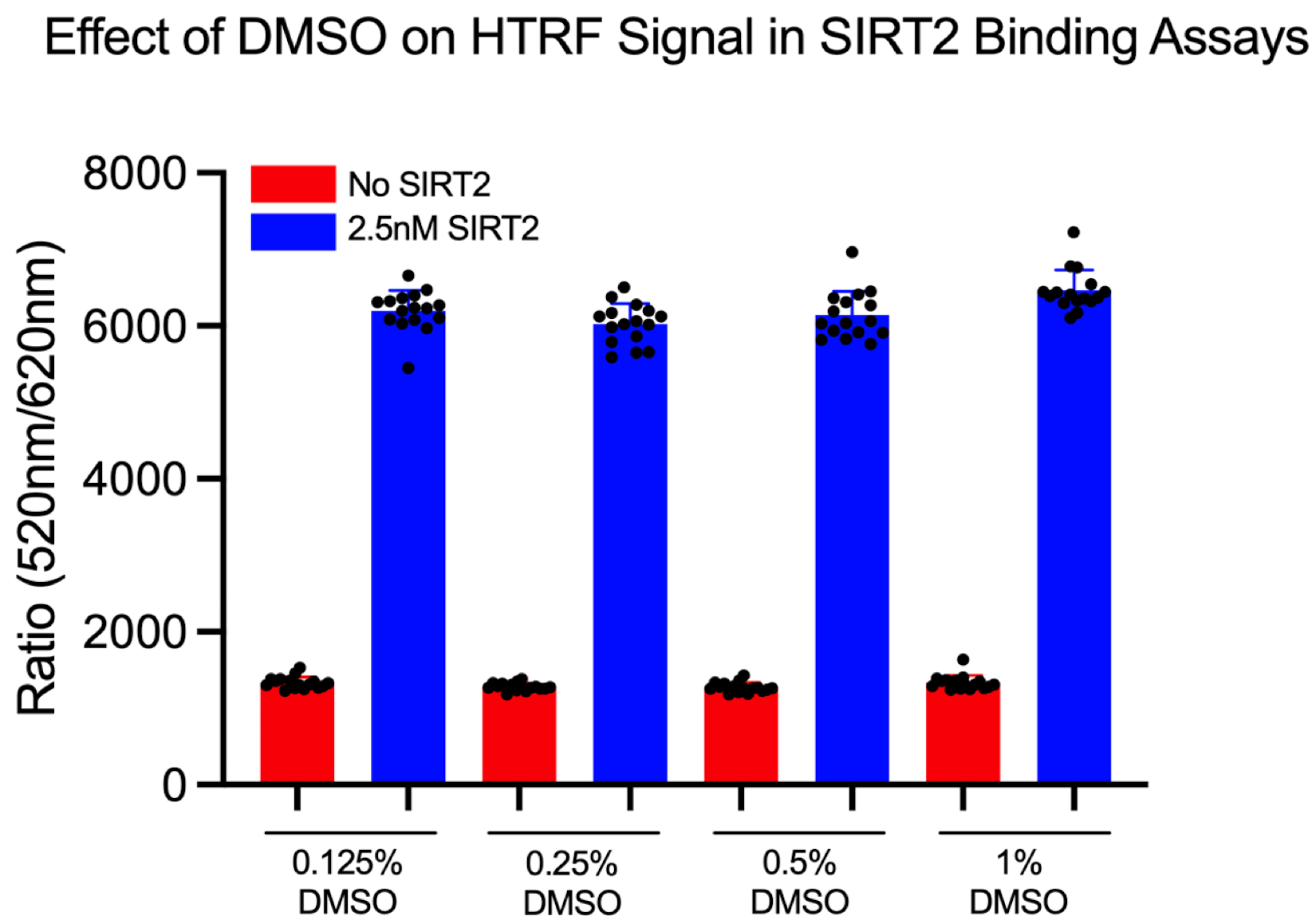

Supplement: S4 Fig — In this experiment, assay conditions were 2.5 nM SIRT2, 0.2 nM terbium cryptate-labeled antibody, and 3 nM FAM-myristoyl-H4K16 peptide. (TIFF) [file pone.0305000.s004.tiff]

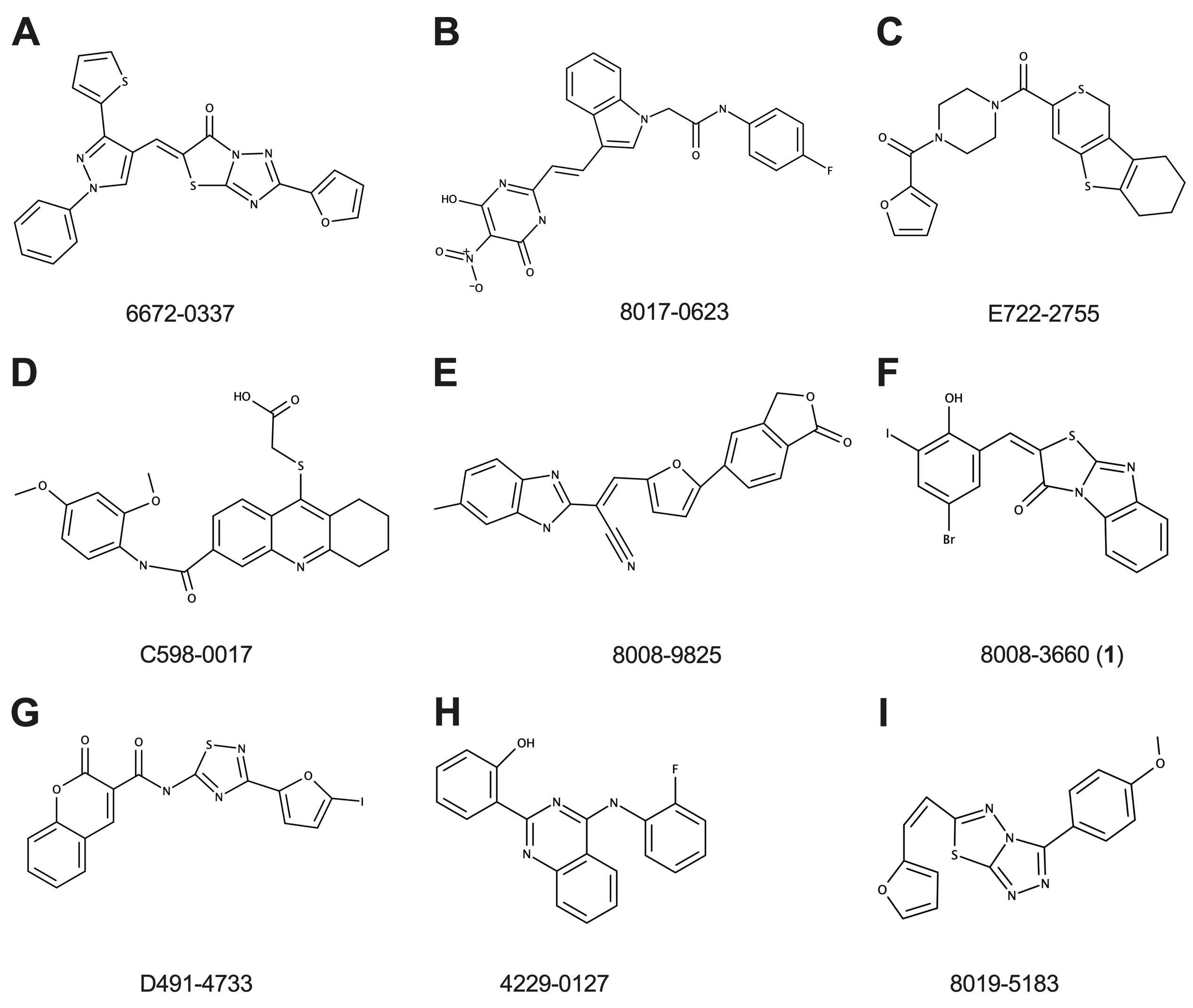

Supplement: S6 Fig — The Compound IDs are also shown in Fig 2A of the main article. (TIFF) [file pone.0305000.s006.tiff]

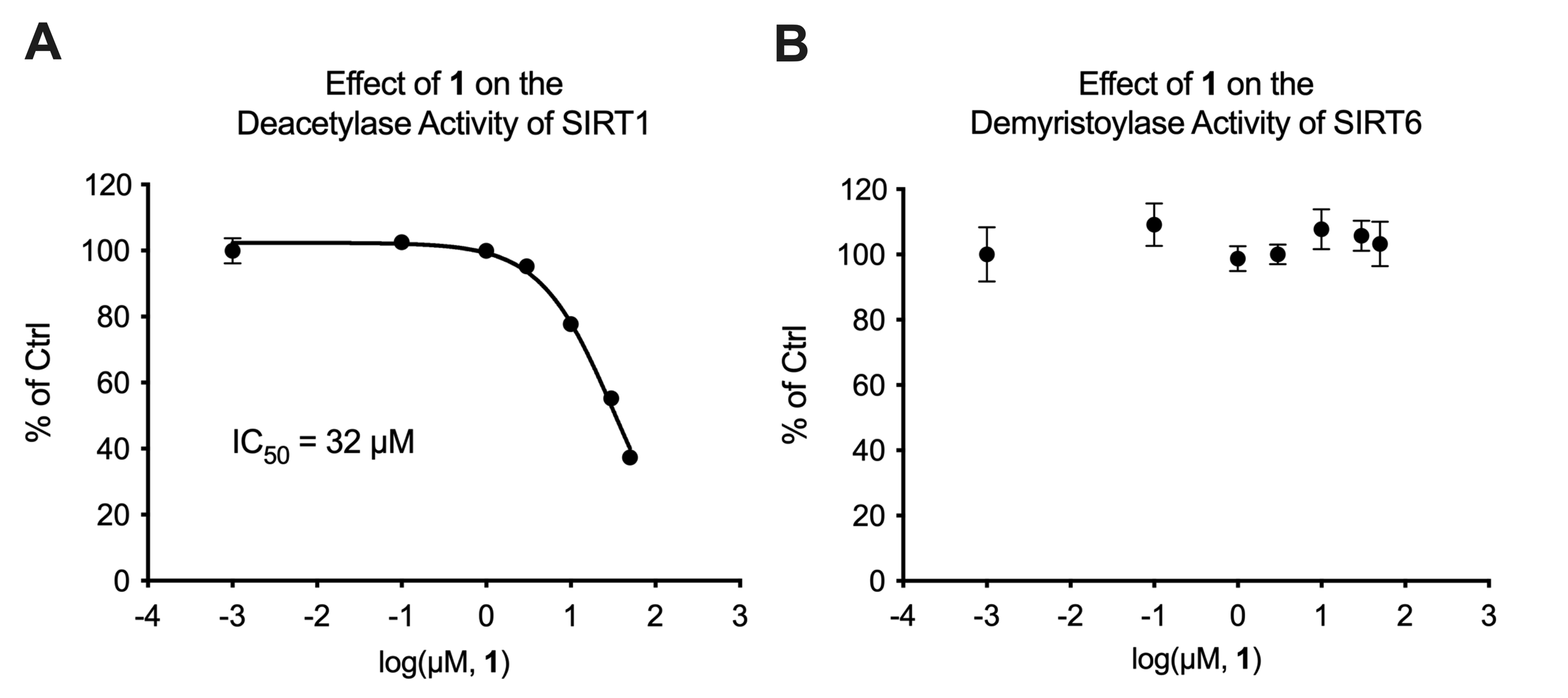

Supplement: S7 Fig — Dose-response relationships showing the effects of 1 on (A) SIRT1 deacetylase activity and (B) SIRT6 demyristoylase activity. (TIFF) [file pone.0305000.s007.tiff]

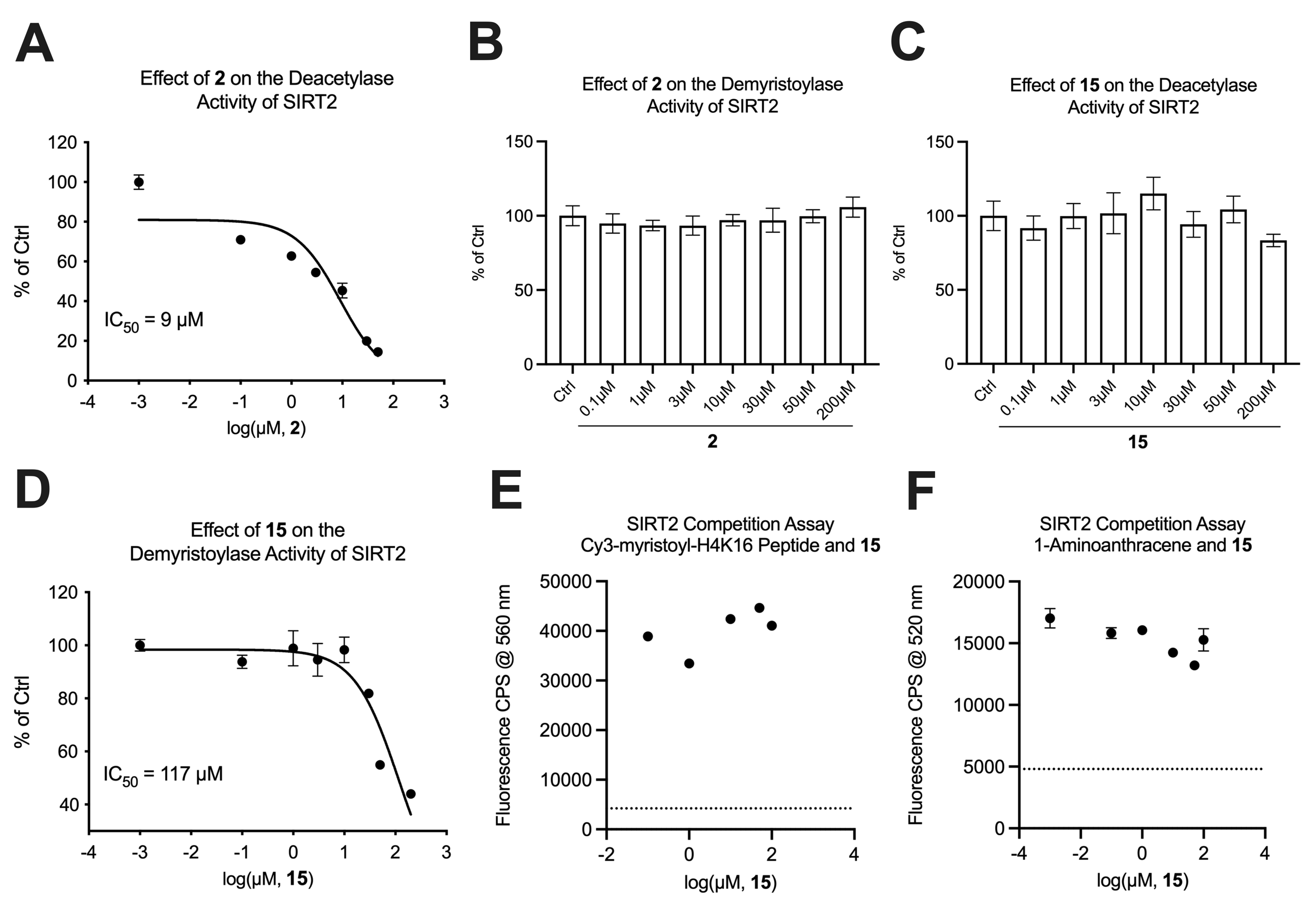

Supplement: S8 Fig — (A) 2 inhibited SIRT2 deacetylase activity with an IC50 of 9 μM. (B) 2 had no effect on SIRT2 demyristoylase activity. (C) 15 had no effect on SIRT2 deacetylase activity. (D) 15 inhibited SIRT2 demyristoylase activity with an IC50 of 117 μM. (E) 15 did not efficiently displace Cy3-myristoyl-H4K16 peptide from SIRT2 at concentrations as high as 100 μM. The dotted line indicates the background fluorescence (peptide only) where 100% inhibition of binding would occur. (F) 15 did not efficiently displace 1-aminoanthracene from SIRT2 at concentrations as high as 100 μM. The dotted line indicates the background fluorescence (1-aminoanthracene only) where 100% inhibition of binding would occur. (TIFF) [file pone.0305000.s008.tiff]

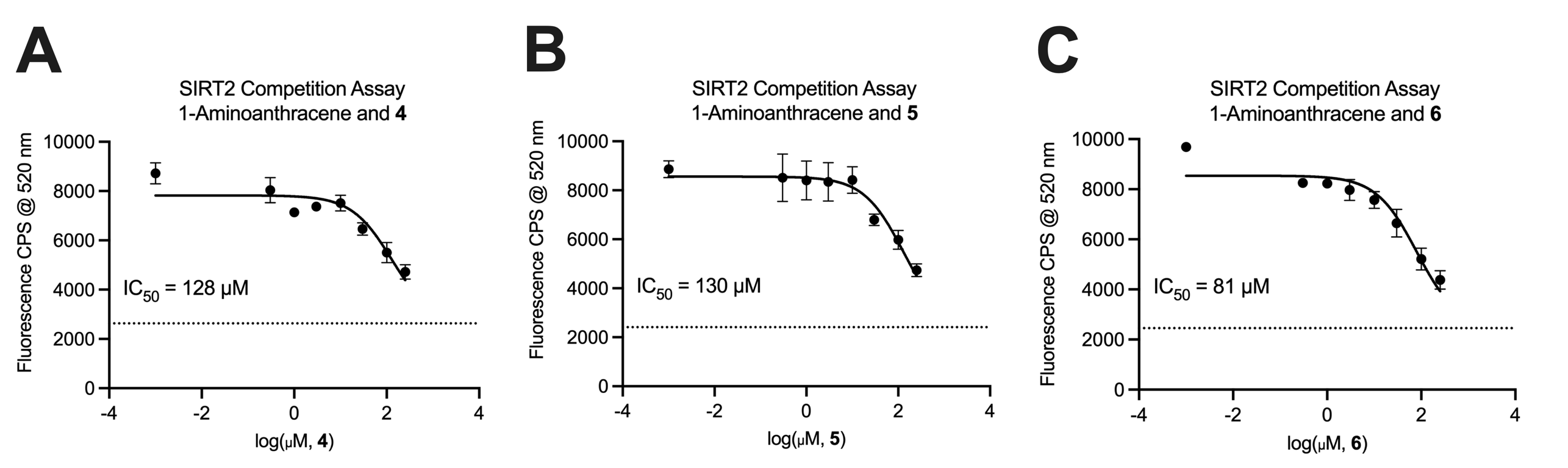

Supplement: S9 Fig — For all assays, the SIRT2 concentration was constant at 4 μM and the 1-aminoanthracene concentration was 100 nM. The dotted line in the panels shows the fluorescence of 1-aminoanthracene alone in the absence of SIRT2, which was the baseline for 100% inhibition of 1-aminoanthracene binding. (A) 4 competed 1-aminoanthracene from SIRT2 with an IC50 of 128 μM, which was used to calculate a Kd of 116 μM for the interaction of 4 with SIRT2. (B) 5 competed 1-aminoanthracene from SIRT2 with an IC50 of 130 μM, which was used to calculate a Kd of 117 μM for the interaction of 5 with SIRT2. (C) 6 competed 1-aminoanthracene from SIRT2 with an IC50 of 81 μM, which was used to calculate a Kd of 73 μM for the interaction of 6 with SIRT2. (TIFF) [file pone.0305000.s009.tiff]

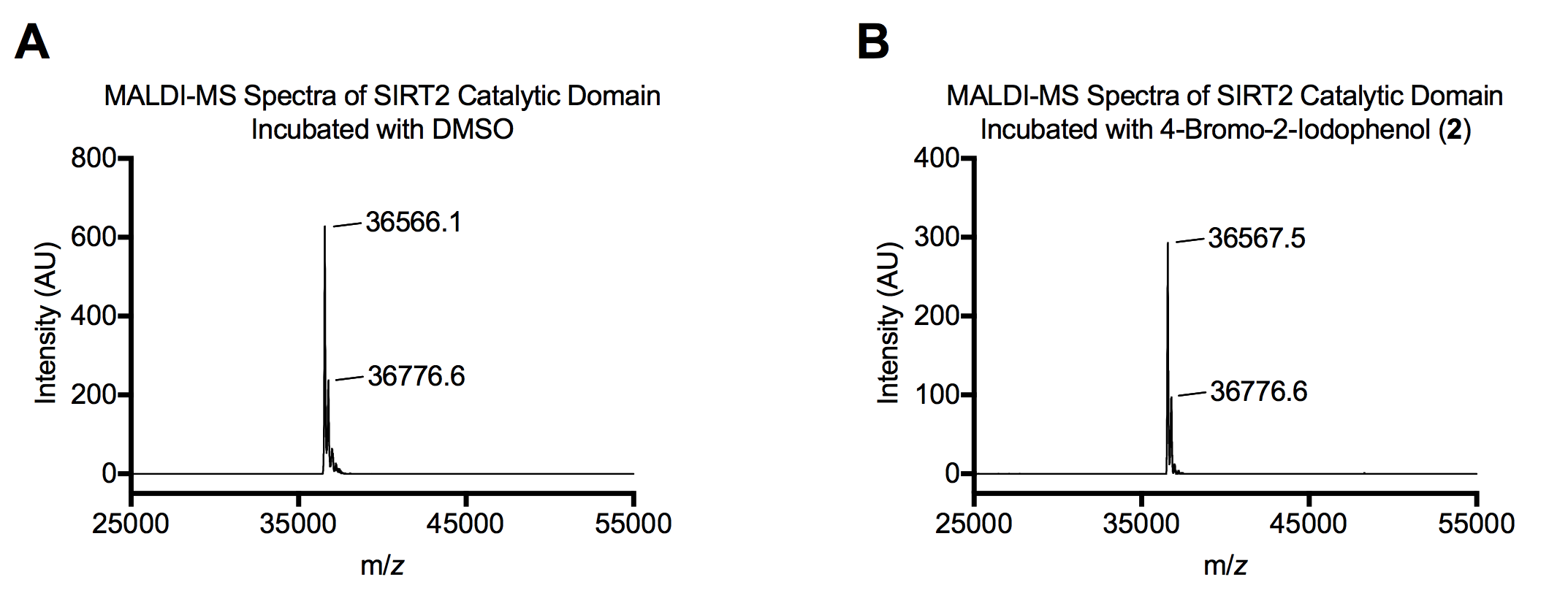

Supplement: S10 Fig — (A) MALDI-MS spectra of 10 μM SIRT2 after incubation in PBS with 1% DMSO at 37°C for 30 minutes. (B) MALDI-MS spectra of 10 μM SIRT2 after incubation with 100 μM of 2 in PBS with 1% DMSO at 37°C for 30 minutes. The predicted monoisotopic mass for this SIRT2 protein was 36552 Da, and the predicted average mass was 36576 Da; thus, the main peaks represented +1 ions in the isotopic cluster. Notably, the observed mass of the protein does not change after incubation with the ligand. The intact protein spectra were acquired with a Bruker microflex instrument in linear, positive ion mode using sinapinic acid as the matrix. Sinapinic acid was dissolved in 50% acetonitrile/50% water with 0.1% trifluoroacetic acid, which was also used to dilute protein prior to spotting on the MALDI plate. (TIFF) [file pone.0305000.s010.tiff]

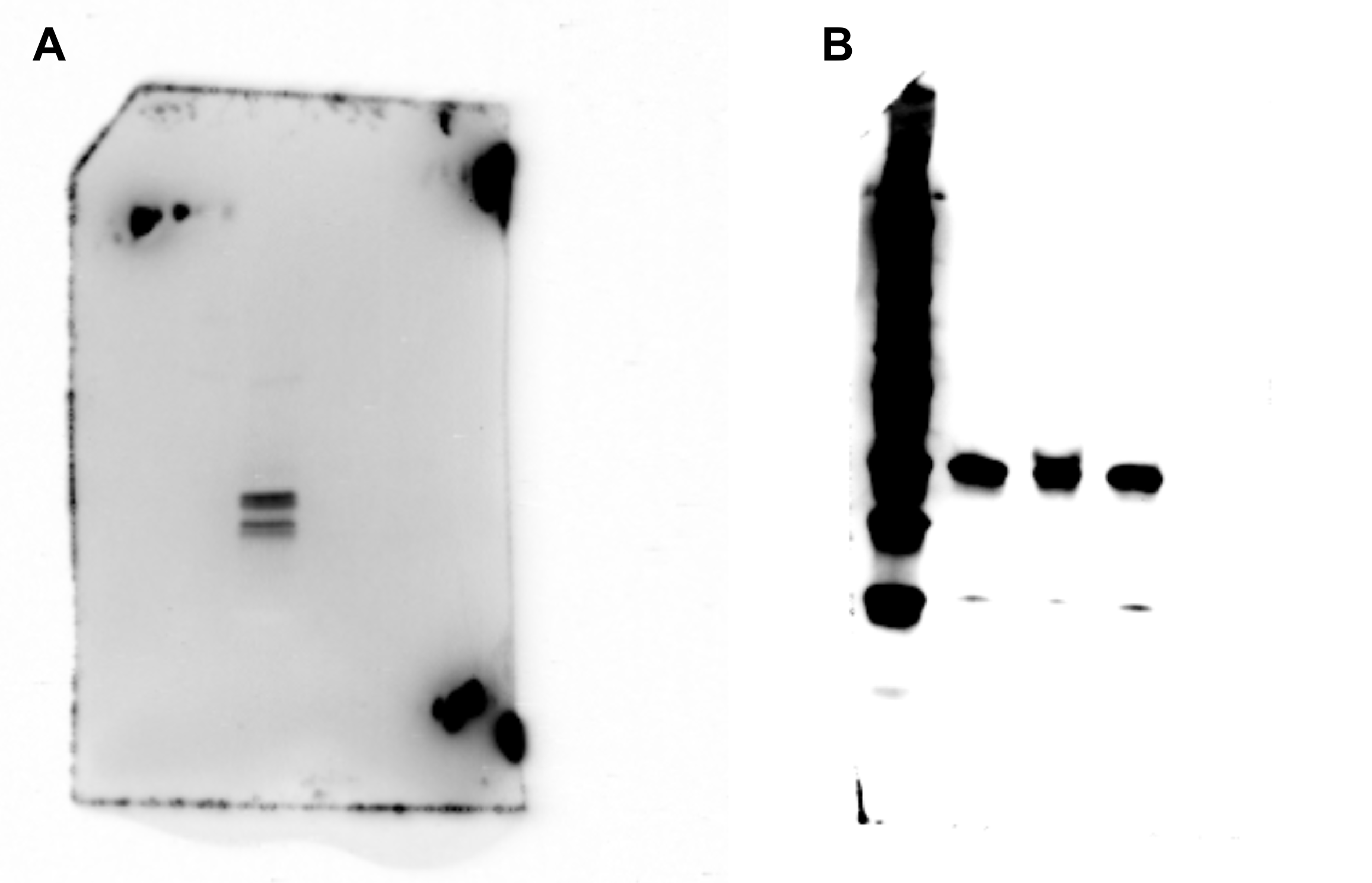

Supplement: S11 Fig — Per journal policy, we provide (A) the original Western blot for SIRT2 and (B) the original Western blot for alpha-tubulin, both of which are shown in Fig 4C. For both blots, the experiment contained a third lane that was cropped out of the main text figure and is not relevant for the reported results. (TIF) [file pone.0305000.s011.tif]
