# Supplementary material for: A homogeneous time-resolved fluorescence screen to identify SIRT2 deacetylase and defatty-acylase inhibitors
Source: PLoS One. 2024 Jun 24;19(6):e0305000. doi: 10.1371/journal.pone.0305000 (PMC11195995; doi:10.1371/journal.pone.0305000)

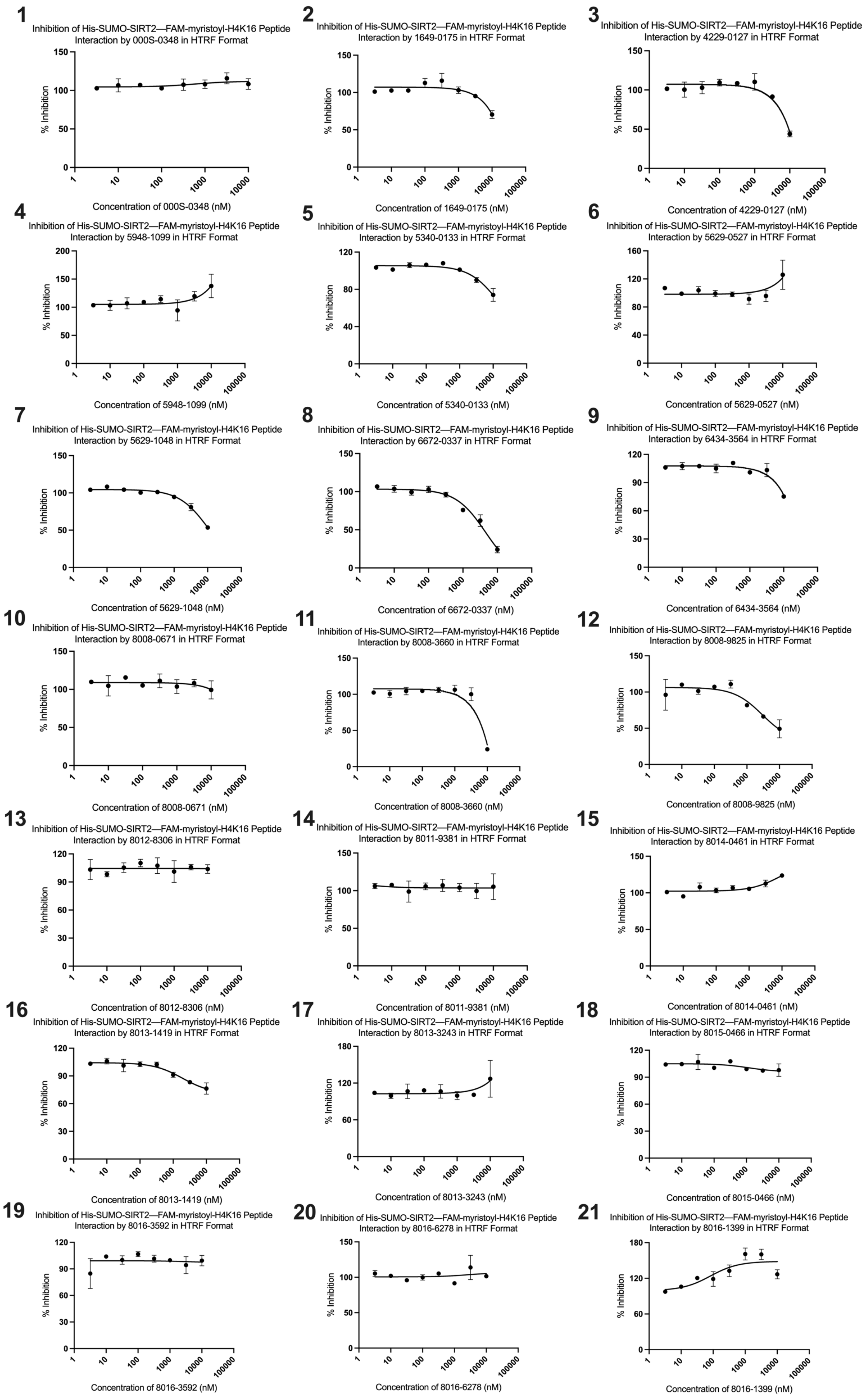
1
Inhibition of His-SUMO-SIRT2—FAM-myristoyl-H4K16 Peptide Interaction by 000S-0348 in HTRF Format
% Inhibition
Concentration of 000S-0348 (nM)
2
Inhibition of His-SUMO-SIRT2—FAM-myristoyl-H4K16 Peptide Interaction by 1649-0175 in HTRF Format
% Inhibition
Concentration of 1649-0175 (nM)
3
Inhibition of His-SUMO-SIRT2—FAM-myristoyl-H4K16 Peptide Interaction by 4229-0127 in HTRF Format
% Inhibition
Concentration of 4229-0127 (nM)
4
Inhibition of His-SUMO-SIRT2—FAM-myristoyl-H4K16 Peptide Interaction by 5948-1099 in HTRF Format
% Inhibition
Concentration of 5948-1099 (nM)
5
Inhibition of His-SUMO-SIRT2—FAM-myristoyl-H4K16 Peptide Interaction by 5340-0133 in HTRF Format
% Inhibition
Concentration of 5340-0133 (nM)
6
Inhibition of His-SUMO-SIRT2—FAM-myristoyl-H4K16 Peptide Interaction by 5629-0527 in HTRF Format
% Inhibition
Concentration of 5629-0527 (nM)
7
Inhibition of His-SUMO-SIRT2—FAM-myristoyl-H4K16 Peptide Interaction by 5629-1048 in HTRF Format
% Inhibition
Concentration of 5629-1048 (nM)
8
Inhibition of His-SUMO-SIRT2—FAM-myristoyl-H4K16 Peptide Interaction by 6672-0337 in HTRF Format
% Inhibition
Concentration of 6672-0337 (nM)
9
Inhibition of His-SUMO-SIRT2—FAM-myristoyl-H4K16 Peptide Interaction by 6434-3564 in HTRF Format
% Inhibition
Concentration of 6434-3564 (nM)
10
Inhibition of His-SUMO-SIRT2—FAM-myristoyl-H4K16 Peptide Interaction by 8008-0671 in HTRF Format
% Inhibition
Concentration of 8008-0671 (nM)
11
Inhibition of His-SUMO-SIRT2—FAM-myristoyl-H4K16 Peptide Interaction by 8008-3660 in HTRF Format
% Inhibition
Concentration of 8008-3660 (nM)
12
Inhibition of His-SUMO-SIRT2—FAM-myristoyl-H4K16 Peptide Interaction by 8008-9825 in HTRF Format
% Inhibition
Concentration of 8008-9825 (nM)
13
Inhibition of His-SUMO-SIRT2—FAM-myristoyl-H4K16 Peptide Interaction by 8012-8306 in HTRF Format
% Inhibition
Concentration of 8012-8306 (nM)
14
Inhibition of His-SUMO-SIRT2—FAM-myristoyl-H4K16 Peptide Interaction by 8011-9381 in HTRF Format
% Inhibition
Concentration of 8011-9381 (nM)
15
Inhibition of His-SUMO-SIRT2—FAM-myristoyl-H4K16 Peptide Interaction by 8014-0461 in HTRF Format
% Inhibition
Concentration of 8014-0461 (nM)
16
Inhibition of His-SUMO-SIRT2—FAM-myristoyl-H4K16 Peptide Interaction by 8013-1419 in HTRF Format
% Inhibition
Concentration of 8013-1419 (nM)
17
Inhibition of His-SUMO-SIRT2—FAM-myristoyl-H4K16 Peptide Interaction by 8013-3243 in HTRF Format
% Inhibition
Concentration of 8013-3243 (nM)
18
Inhibition of His-SUMO-SIRT2—FAM-myristoyl-H4K16 Peptide Interaction by 8015-0466 in HTRF Format
% Inhibition
Concentration of 8015-0466 (nM)
19
Inhibition of His-SUMO-SIRT2—FAM-myristoyl-H4K16 Peptide Interaction by 8016-3592 in HTRF Format
% Inhibition
Concentration of 8016-3592 (nM)
20
Inhibition of His-SUMO-SIRT2—FAM-myristoyl-H4K16 Peptide Interaction by 8016-6278 in HTRF Format
% Inhibition
Concentration of 8016-6278 (nM)
21
Inhibition of His-SUMO-SIRT2—FAM-myristoyl-H4K16 Peptide Interaction by 8016-1399 in HTRF Format
% Inhibition
Concentration of 8016-1399 (nM)

22

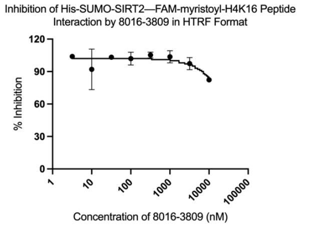

23

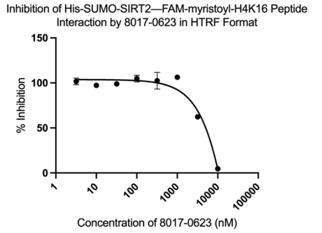

24

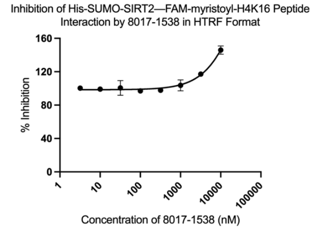

25

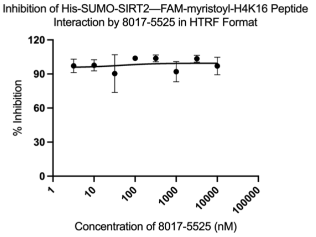

26

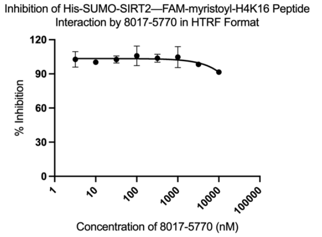

27

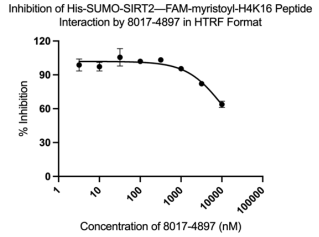

28

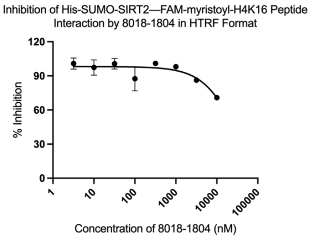

29

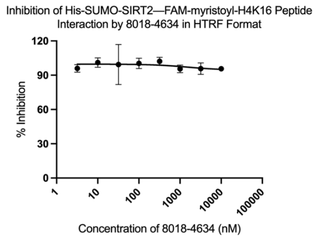

30

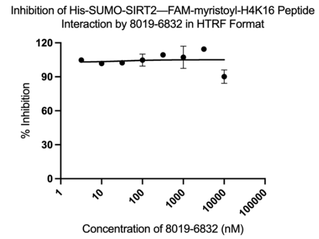

31

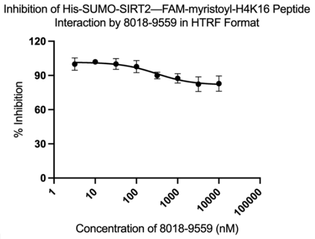

32

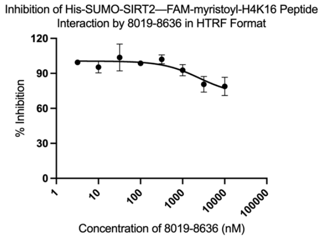

33

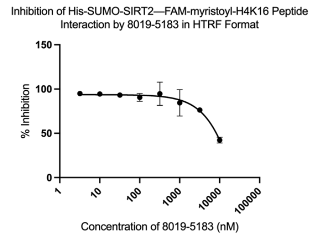

34

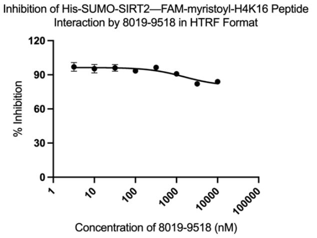

35

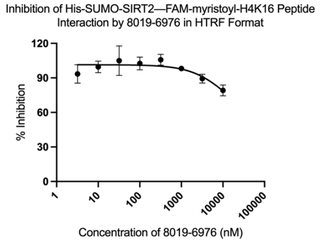

36

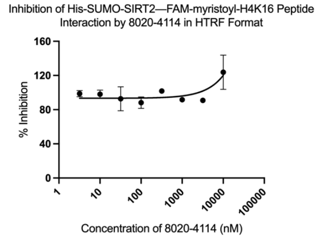

37

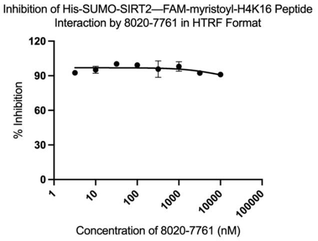

38

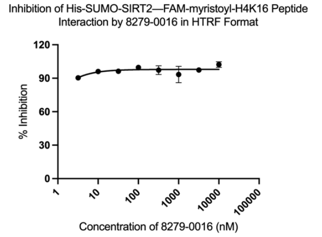

39

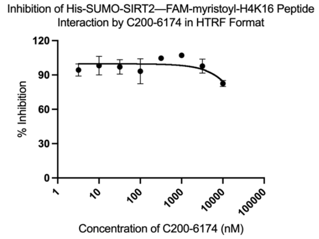

40

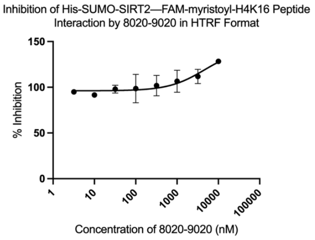

41

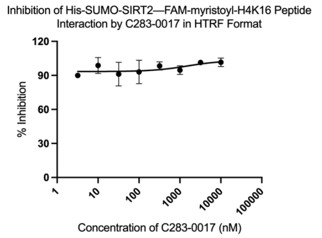

42

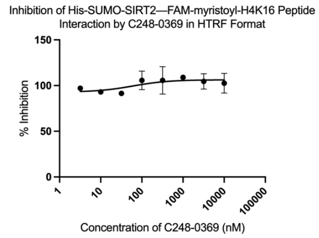

43

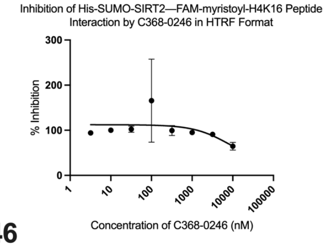

44

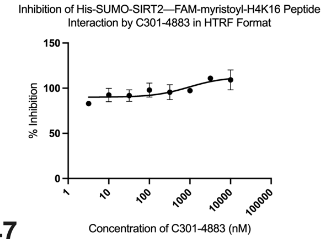

45

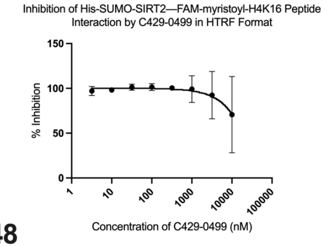

46

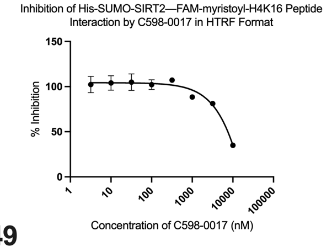

47

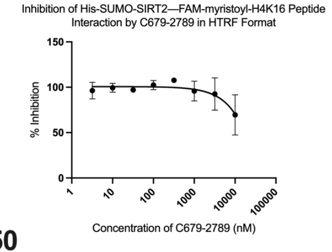

48

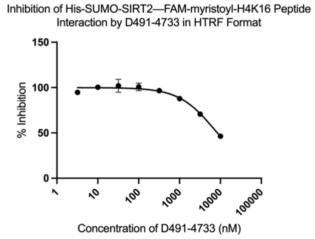

49

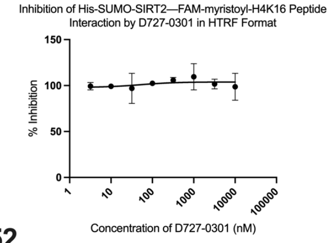

50

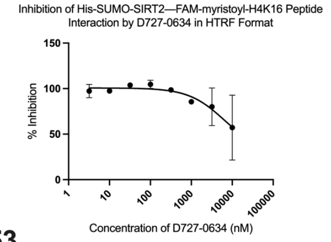

51

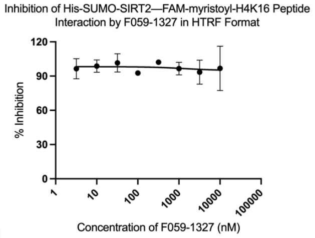

52

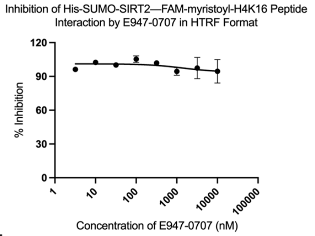

53

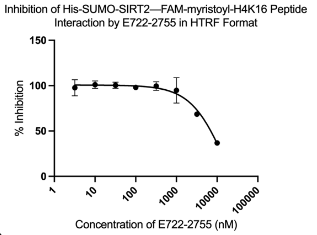

54

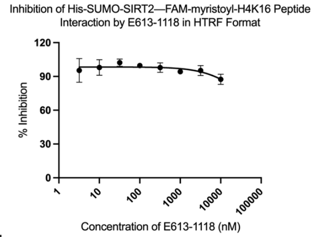

55

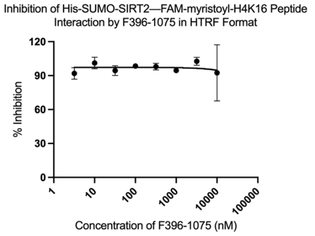

56

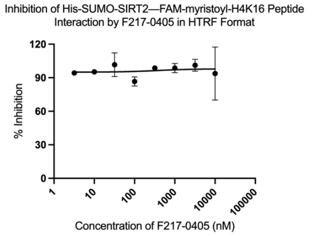

57

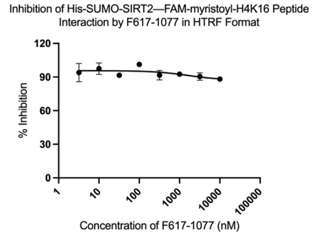

58

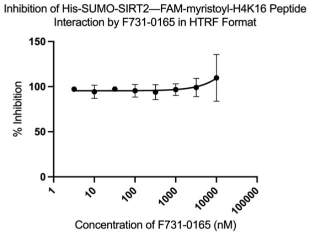

59

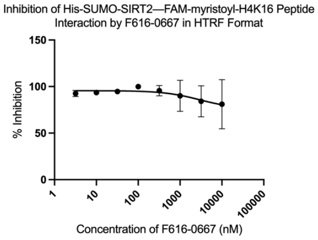

60

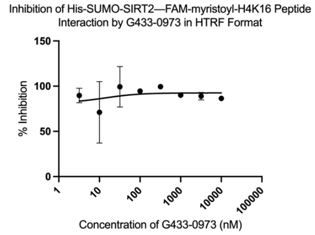

61

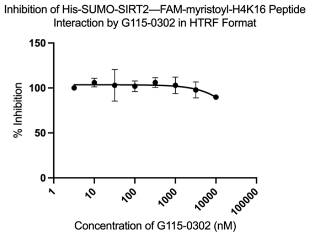

62

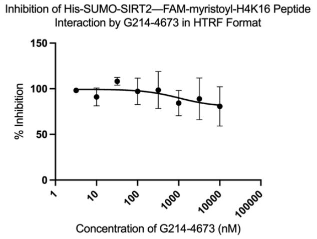

63

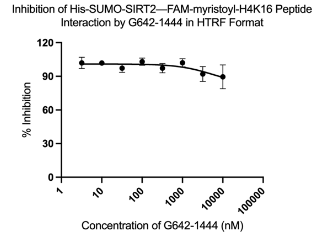

Supplement: S5 Fig — (PDF) [file pone.0305000.s005.pdf]
