# Supplementary material for: A homogeneous time-resolved fluorescence screen to identify SIRT2 deacetylase and defatty-acylase inhibitors
Source: PLoS One. 2024 Jun 24;19(6):e0305000. doi: 10.1371/journal.pone.0305000 (PMC11195995; doi:10.1371/journal.pone.0305000)
Supplement: S1 Table — (PDF) [file pone.0305000.s012.pdf]

S1 Table. Mass, Volume, and Polarity of Investigated Compounds

Compound	Structure	Mass (Da)[a]	Polar Surface Area (cm^2)[a]	van der Waals volume (Å^3)[a]	Calculated logP[a]
**1** (8008-3660)	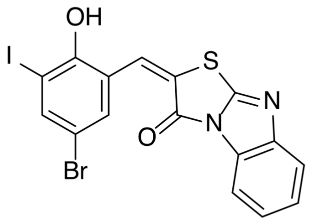	499.12	55.12	279.82	4.89
**2** **(**4-bromo-2-iodophenol)	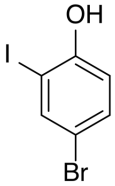	298.90	20.23	133.23	3.61
**3** (2-6-diisopropylphenol) (propofol)	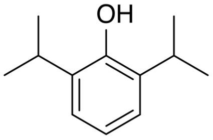	178.27	20.23	192.49	4.20
**4** (2-isopropyl-5-methylphenol) (thymol)	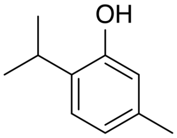	150.22	20.23	158.39	3.46
**5** 5-isopropyl-2-methylphenol (carvacrol)	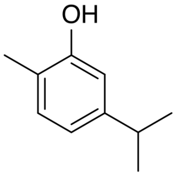	150.22	20.23	158.32	3.46
**6** 5-methyl-2-pentylphenol (amylmetacresol)	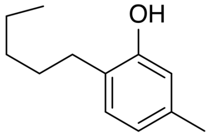	178.27	20.23	192.14	4.60
**7** *meta*-azi-propofol	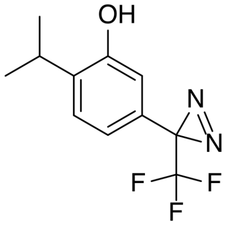	244.21	44.95	196.63	4.01
**8** 3-phenyl-3-(trifluoromethyl)-3H-diazirine	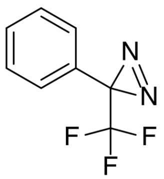	186.13	24.72	136.97	3.13

**9**	4-bromo-2-fluoro-6-iodophenol	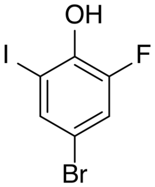	316.89	20.23	138.09	3.73
**10**	4-bromo-2,6-diiodophenol	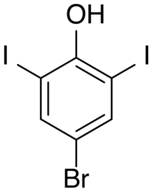	424.80	20.23	157.69	4.73
**11**	4-bromo-2-(trifluoromethyl) phenol	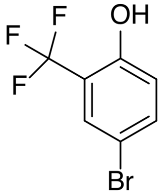	241.00	20.23	140.64	3.37
**12**	4-bromo-2,6-difluorophenol	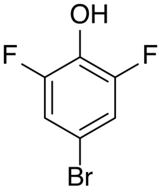	208.99	20.23	118.48	2.74
**13**	4-butoxy-2,6-difluorophenol	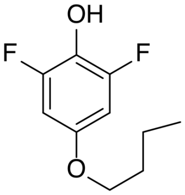	202.20	29.46	177.17	3.21
**14**	2-fluoro-5-(trifluoromethyl) phenol	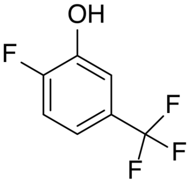	180.10	20.23	127.20	2.71
**15**	[1,3]thiazolo[3,2-a]benzimidazole-3(2H)-one	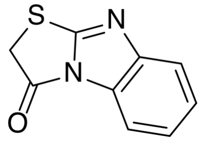	190.22	34.89	148.68	0.96

[a]Calculated using MarvinSketch version 14.7.14.0 (ChemAxon). The calculated logP (octanol/water partition coefficient) was determined using the model by Klopman (https://doi.org/10.1021/ci00020a009).
